# Community-based actions in consulates: a new paradigm for opportunities for systematic integration in Chagas disease detection

**DOI:** 10.1186/s12879-023-08844-2

**Published:** 2023-12-01

**Authors:** Jordi Gómez i Prat, Maria Serrano Gregori, Isabel Claveria Guiu, Estefa Choque, Maria Delmans Flores-Chavez, Israel Molina, Francesc Zarzuela, Elena Sulleiro, Aurore Dehousse, Pedro Albajar-Vinas, Hakima Ouaarab

**Affiliations:** 1 Department of Infectious Diseases, Public Health and Community Team (eSPiC), Drassanes-Vall d’Hebron International Health Unit (USIDVH), Vall d’Hebron University Hospital, Barcelona, Spain; 2 International Health Program of the Catalan Institute of Health (PROSICS), Barcelona, Spain; 3 Association of Friends of People With Chagas Disease - ASAPECHA2 International Health Program of the Catalan Institute of Health (PROSICS), Barcelona, Spain; 4 Mundo Sano Foundation, Barcelona, Spain; 5 Leishmaniasis and Chagas Disease Unit, National Centre for Microbiology, Instituto de Salud Carlos III. Madrid, Barcelona, Spain; 6 Department of Infectious Diseases, Tropical Medicine Unit, Vall d’Hebron University Hospital, Barcelona, Spain; 7 Department of Microbiology, Tropical Medicine Unit, Vall d’Hebron University Hospital, PROSICS Barcelona, Barcelona, Spain; 8 Department of Control of Neglected Tropical Diseases, World Health Organization, Geneva, Switzerland

**Keywords:** Chagas disease, *In-situ* screening, Health community-based strategy, Concomitant infections, Migrant population, Opportunities for systematic integration

## Abstract

Research has shown that multidimensional approaches to Chagas disease (CD), integrating its biomedical and psycho-socio-cultural components, are successful in enhancing early access to diagnosis, treatment and sustainable follow-up.

For the first time, a consulate was selected for a community-based CD detection campaign. Two different strategies were designed, implemented and compared between 2021 and 2022 at the Consulate General of Bolivia and a reference health facility in Barcelona open to all Bolivians in Catalonia.

Strategy 1 consisted in CD awareness-raising activities before referring those interested to the reference facility for infectious disease screening. Strategy 2 offered additional *in-situ* serological CD screening. Most of the 307 participants were Bolivian women residents in Barcelona. In strategy 1, 73 people (35.8% of those who were offered the test) were screened and 19.2% of them were diagnosed with CD. Additionally, 53,4% completed their vaccination schedules and 28.8% were treated for other parasitic infections (strongyloidiasis, giardiasis, eosinophilia, syphilis). In strategy 2, 103 people were screened *in-situ* (100% of those who were offered the test) and 13.5% received a CD diagnosis. 21,4% completed their vaccination schedule at the reference health facility and 2,9% were referred for iron deficiency anemia, strongyloidiasis or chronic hepatitis C.

The fact that the screening took place in an official workplace of representatives of their own country, together with the presence of community-based participants fueled trust and increased CD understanding. Each of the strategies assessed had different benefits. Opportunities for systematic integration for CD based on community action in consulates may enhance early access to diagnosis, care and disease prevention.

## Background

Six to seven million people worldwide are estimated to be infected with Chagas disease (CD), an infection caused by the *Trypanosoma cruzi (T. cruzi)* parasite. It is mainly an asymptomatic or oligosymptomatic condition (making it a so-called silent disease), either in the acute or chronic phase of the disease. Nevertheless, without early diagnosis and treatment, one third of the people can deal with a life-threatening condition, affecting mainly the heart, but also the digestive and neurological systems. Since 2005 it has been part of the World Health Organization (WHO) list of neglected tropical diseases (NTDs), often referred to as “silenced diseases” with little political attention received. As such, and despite cases having already been detected in forty four countries of five continents, the proportion of people who have been already diagnosed and treated has been estimated at less than 10% worldwide [1, 2].

Most CD cases outside Latin America are the result of population movements between the region and other parts of the world, including migrants and travelers. Fear and various stigmas attached to CD, along with administrative barriers, cause substantial difficulties for the migrant population to access public healthcare systems [3–7]. As Sanmartino et al. remind us, “people at risk of having been infected often prefer not to know whether they have CD, because they are afraid of it and they imagine consequences, owing to popular beliefs or experience of family or friends who did not respond to treatment and/or died”.

The biomedical and psycho-socio-cultural components of the multidimensional [8] understanding are equally fundamental to overcome barriers to early access to diagnosis, treatment and sustainable follow up. They bring in new perspectives, which have helped build new paradigms and methods for the management of CD in a more multidisciplinary and unified way, as explained by Sanmartino et al. [8]. Indeed, such components are key to confront the diversity of challenges faced by people affected with CD in different cultural and social contexts and eliminate the psycho-social barriers that still characterize CD [5, 7, 8]. In recent years, multidimensional materials, tools and strategies of information, education, and communication (IEC) have proven to be crucial for understanding and addressing the public health problem of CD.

In the last decade, the organizations of people affected by CD spread worldwide—so far thirty two in fourteen countries—and they are one of the most transformative and impactful initiatives in terms of IEC. They are all part of the International Federation of Associations of People Affected by Chagas Disease (FINDECHAGAS) [9], which since 2010 has inspired the objectives of the associations, one of the main being the collaboration with national health systems, regional and international organizations to improve access to diagnosis and treatment. This is particularly difficult to achieve in areas where CD is unknown, or is not considered as a public health issue. That is often the case in urban and non-endemic areas. IEC has shown to be fundamental in detecting new cases in those contexts. Community *in-situ* screening events have been implemented in a way that allows the team work between peer health educators from the CD-affected associations and healthcare professionals from different institutions [10–12]. The peer educators, some of whom were members of the Association of Friends of People with Chagas Disease (ASAPECHA) in Barcelona, were trained with programs that aim to spread awareness of CD [13].

The Public Health and Community team (eSPiC), part of the Drassanes-Vall d’Hebron International Health Unit (USIDVH) of the International Health Program of the Catalan Institute of Health (PROSICS), has worked with ASAPECHA, in Barcelona, since 2008. Together they have been implementing interventions to foster detection and access to CD diagnosis and treatment, as well as conducting *in-situ* screenings since 2014 for both people from the Latin American community or people at risk of infection—notably people who traveled to endemic areas [11]. The same year, the eSPiC also started what is now a strong partnership with the Consulate General of Bolivia in Barcelona, since the Bolivian community has a historically high rate of *T. cruzi* infection.

In 2020, the eSPiC, with the help of ASAPECHA, carried out a research that showed that systematic and multidimensional approaches of CD have positive results on the diagnosis rate [14]. Based on that, between January 2021 and March 2022 it conducted another community-based research within the premises of the Consulate General of Bolivia in Barcelona. It targeted Bolivian citizens who were simply going to the consulate for administrative matters to carry out a screening campaign. Building on two previously-validated strategies [15], it included opportunities for systematic integration (OSI) to look at some possible coinfections. Those correspond to using the screening of an infection as an opportunity to carry out an additional test for a different condition. Progressively implemented since 2007, OSI offer the possibility to increase detection, diagnosis confirmation, care, prevention, control and cost-effectiveness [14].

Drawing on all those elements, this research aimed at designing and comparing two outreach strategies within an innovative space—a consulate—to increase the diagnosis coverage of infected people with *T. cruzi* within the Bolivian population of Catalonia.

This research will aim at answering the following questions: in the context of a European country, such as Spain, what role could an official building of a foreign government known to have one of the highest number of cases in the world have in the detection of CD? More specifically, how can community-based actions led in a consulate improve CD detection rates and fuel opportunities of systematic integration in the detection of *T. cruzi* infection in countries outside Latin America?

In an attempt to answer these questions, we made the following assumptions: 1) The consulate constituted an ideal place to carry out those interventions since a wide array of people (both men and women, all ages combined) needs to go there for administrative matters 2) the involvement of both the community health agents of Bolivian origin and the consulate staff in the awareness raising process, speaking Spanish with the same accent of the target population as well as Quechua and other dialects from Bolivia, laid the foundation for an environment of trust and facilitated the participation of the people in the research 3) in situ screening (strategy 2) would have a greater reach, and give access to diagnosis to people that do not live in Barcelona or at least not next to a health facility; 4) giving access to CD screening and diagnosis in the health facility (strategy 1) represents an opportunity for systematic integration to diagnose concomitant infections and assess other health conditions.

## Methods

### Intervention design, population, and sample

The research was performed between 2021 and 2022 at the Consulate General of Bolivia in Barcelona and at the eSPiC of the USIDVH. It was open to anyone who was willing to get screened and was made possible by the long lasting partnership between the Bolivian consulate and eSPiC, which have been collaborating on CD awareness raising projects since 2014. Due to the high prevalence of *T. cruzi* infection within the Bolivian population [1, 16] and the fact that Catalonia counts 23,840 Bolivians [17]—and that is not counting those who were naturalized Spanish—the consulate is eager to promote screening interventions and help increasing diagnosis and promoting access to healthcare through such initiatives.

Prior to this research, the consent of the Ministry of Health of Bolivia was requested by the Consulate General in Barcelona. The research was also discussed with every employee of the consulate, in order to ensure a robust collaboration and efficient communication between people on site. Both strongly relied on community health agents (CHA), mostly from ASAPECHA, who had been previously trained on the comprehensive understanding of the disease, developing a specific way to address the psycho-social barriers to CD diagnosis. They acted as a bridge between health professionals team members and the Bolivian community by conducting patient outreach and aiding in the diagnosis-treatment process. The discussions were facilitated by the fact that most CHA were Bolivians. People that were targeted could hence easily understand them since they shared the same language (Spanish, Quechua…), accent and wording, which ultimately fueled trust between them.

Community health actions were performed weekly. Strategy 1 consisted in raising awareness on CD within the premises of the consulate three days a month: educational materials were projected on a screen of the waiting room and community health agents were present to discuss with people about CD, explaining what it is, how it is diagnosed and treated. Those who were interested in being tested for CD were given appointments at the eSPiC to do an infectious disease screening and health assessment. A total of 33 awareness and referral interventions were performed at the consulate during 12 months (from January to December 2021). In Strategy 2, additionally to the IEC components of the Strategy1, *in-situ CD* screenings were performed directly at the consulate. After being informed about CD by the community health agent, those interested in participating in the screening were attended by healthcare professionals (nurses and physicians) in a designated area of the consulate. The collected samples were transported and analyzed at the microbiology laboratory. Those who tested negative for *T. cruzi* infection were informed by phone, while those who tested positive were given an appointment at USIDVH, where they confirmed their diagnosis and received a CD treatment plan, together with an infectious diseases screening and health assessment. A total of six *in-situ* interventions were performed from June 2021 to February 2022, alternately with Strategy 1. In the case of strategy 1, where people would go to the premises of the USIDVH, people potentially at risk—who recently migrated from Bolivia and people returning from a trip visiting-friends and relatives were also offered an infectious disease screening according to the health facility’s protocol [18].

*T. cruzi* infection diagnosis of the enrolled patients was established based on the results of two different assays for IgG anti-*T. cruzi* [19]: serum samples were tested by an electrochemiluminescence immunoassay (Elecsys Chagas, Roche Diagnostics, Manheim, Germany) and those with a positive result were subsequently analyzed using a commercial ELISA (Ortho *T. cruzi* ELISA, Johnson & Johnson, High Wycombe, United Kingdom). Infection was confirmed when the serum sample was positive for both essays. Patients who had a confirmed result went through a general assessment and were offered antiparasitic treatment and follow-up.

### Data collection

In order to have a comprehensive understanding of the socio-demographic background of the participants, we collected the following information: gender, age, country of origin, place of residence, time of residence in Spain, financial situation, administrative situation, and whether or not they had an individual health card. Here it is important to stress that in the framework of this research, people had access to the CD screening no matter if they carried the card or not. In Strategy 1 this information was collected during an individual interview by the community health agent at the consulate. In Strategy 2 data was collected through phone interviews.

From February to April 2022, a first review of the medical histories of the participants was performed, with prior consent, in order to fill in missing information and to include more information related to the CD screening (diagnosis, symptoms, treatment, and monitoring) and the general preventative screening (other healthcare needs that were addressed).

### Statistical analysis

For each strategy, an analysis of the socio-demographic variables relating to the total number of participants was performed, together with the number of screenings and the number of people diagnosed with CD. This enabled the distribution of each variable and the differences between groups to be determined. A random distribution was assumed for values missing from the socio-demographic variables (missing completely at random – MCAR) occurring due to the difficulties of data collection by telephone interviews. Another descriptive analysis was performed on the results of the CD screening, the infectious disease screening and health assessment, also separated by strategy. In order to compare the prevalence rates of CD found with each strategy, both the raw and adjusted prevalence rate ratios were calculated for both groups (with confidence intervals of 95%) through Poisson regression models with robust variance, using generalized linear models and adjusting for age and sex^1^,^2^.

### Ethical considerations

Review Board approvals (PR(AG)371/2019) were obtained from the Ethics Committee of the Vall d'Hebron Research Institute, according to the principles expressed in the Declaration of Helsinki. Prior to participating, all participants received oral information of the intervention prior and gave oral informed consent. For subjects under 16 and illiterate, parents or legal guardians gave oral informed consent to participate. The oral informed consent was approved by Clinical Research Ethics Committee (CEIm) at the Vall d’Hebron University Hospital. All patient data was codified and analyzed anonymously.

## Results

### Socio-demographic characteristics of participants

The socio-demographic characteristics of those who participated in the screening are shown in Table 1. Out of 307 total participants, the majority were women (63.5%), of Bolivian origin (97.4%), and residents of Barcelona (91.8%). There was a homogenous distribution across age groups, with the 40-to-49 year-old group representing the majority, with 32.9%, almost a third of all participants. Most participants had stable employment (68.4%); 73.3% had arrived in Spain five or more years ago, 87.8% had a Spanish national identity document, and 97% had an individual health card at the time of the intervention.

**Table 1:** Socio-demographic characteristics of participants

	**Total**	**Strategy** **1** (**people** **reached**)	**Strategy** **1** (**people** **screened**)	p1	**Strategy** **2** (***in-situ*** **screening**)	p2	p3
	***N*** = 307	***N*** = 204	***N*** = 73		***N*** = 103		
	***n*** (%)	***n*** (%)	***n*** (%)		***n*** (%)		
**Gender**							
Female	195 (63.5%)	135 (66.2%)	50 (68.5%)	0.60	60 (58.3%)	0.17	0.17
**Age**				0.40		0.089	0.73
19 and under	22 ( 7.2%)	20 ( 9.8%)	4 ( 5.5%)		2 ( 1.9%)		
20–29	37 (12.1%)	24 (11.8%)	10 (13.7%)		13 (12.6%)		
30–39	74 (24.1%)	51 (25.0%)	18 (24.7%)		23 (22.3%)		
40–49	101 (32.9%)	66 (32.4%)	22 (30.1%)		35 (34.0%)		
50 and over	73 (23.8%)	43 (21.1%)	19 (26.0%)		30 (29.1%)		
Country of origin*				1.00		1.00	1.00
Bolivia	299 (97.4%)	199 (97.5%)	71 (97.3%)		100 (97.1%)		
Other	8 ( 2.6%)	5 ( 2.5%)	2 ( 2.7%)		3 ( 2.9%)		
Place of residence*				1.00		** < 0.001**	**0.007**
Barcelona	247 (91.8%)	161 (97.0%)	53 (98.1%)		86 (83.5%)		
Other provinces	22 ( 8.2%)	5 ( 3.0%)	1 ( 1.9%)		17 (16.5%)		
**Years since arrival in Europe**				0.92		0.24	0.31
Less than 3 years	26 ( 9.3%)	16 ( 8.0%)	5 ( 7%)		10 (13%)		
Between 3 and 5 years	49 (17.4%)	39 (19.4%)	14 (19%)		10 (13%)		
5 or more years	206 (73.3%)	146 (72.6%)	53 (74%)		60 (75%)		
**Economic situation**				0.84		0.052	0.22
Stable employment	188 (68.4%)	140 (69.7%)	49 (67%)		48 (64.9%)		
Unstable employment	20 ( 7.3%)	10 ( 5.0%)	4 ( 5%)		10 (13.5%)		
Unemployed	67 (24.4%)	51 (25.4%)	20 (27%)		16 (21.6%)		
**Administrative situation**				0.69		**0.046**	0.29
National identity document/foreigner identity number	244 (87.8%)	183 (90.1%)	65 (89%)		61 (81.3%)		
Passport	34 (12.2%)	20 ( 9.9%)	8 (11%)		14 (18.7%)		
Public healthcare registration card*	292 (97.0%)	201 (98.5%)	71 (97%)	0.26	91 (93.8%)	**0.025**	0.19

For the variables "Place of Residence", "Years Since Arriving in Europe", "Economic Situation", and "Administrative Situation" the percentage of missing values was between 8 and 10%

*P*- value 1: socio-demographic differences between people screened and people not screened (strategy 1)

*P*-value 2: differences between people who participated in strategy 1 and strategy 2

*P*-value 3: differences between people screened in strategy 1 and strategy 2

*P*-values calculated with Chi-squared test except for the variables "Country of Origin", "Place of Residence", and "Public Healthcare Registration Card", which were calculated using Fisher’s exact test

In general, the socio-demographic characteristics were similar among the participants in both strategies. In Strategy 1 no significant differences in socio-demographic characteristics were observed between those who agreed to be screened and those who declined.

Significant statistical differences were observed, however, in the variables “Place of Residence”, “Administrative Situation”, and “individual health card” between the two strategies. In Strategy 1, the percentage of people who did not reside in the province of Barcelona was 3.0%, while in Strategy 2 it was 16.5%. With regard to administrative situations, 9.9% of participants reported having only a passport of his country of origin (without Spanish residency documents—individual health card and identity number for foreign nationals) in Strategy 1, while 18.7% reported having a passport of country of origin in Strategy 2. In Strategy 1, 98.5% of people reported having an individual health card, while 93.8% had one in Strategy 2. People who agreed to undergo screening were mostly residents in Catalonia; in Strategy 1, 1.9% of the participants lived outside of Barcelona, in contrast to 16.5% in Strategy 2.

### Results of the CD screening

**Fig. 1:**
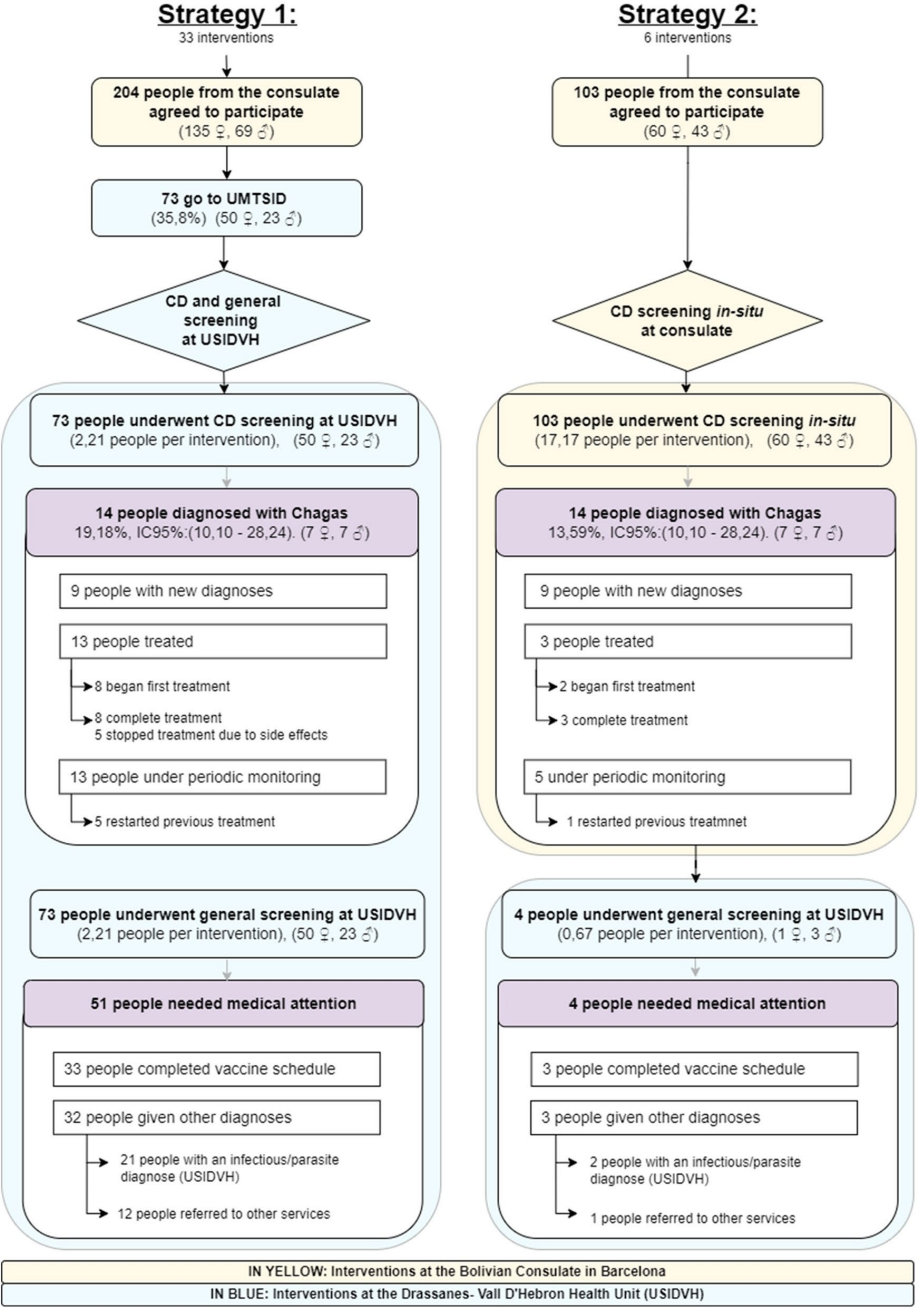
Primary results of both strategies

The first part of Fig. 1 describes the first salient results of CD screenings in both strategies.

Out of the 204 people invited for screening in Strategy 1 during the 33 interventions performed at the consulate, 73 were screened at USIDVH,—35.8%—corresponding to six people sensitized and 2.2 people screened per intervention performed. Among those screened, 14 people were diagnosed with CD (19.8% (IC95%: 10.10–28.24)). Nine of them were diagnosed for the first time. The majority of those diagnosed had CD in a chronic phase and did not present any related symptoms; chronic CD cardiac alterations were detected in only one patient. Of the 14 people diagnosed with CD, 13 began a periodic follow-up plan after the intervention. Five of those people had been previously diagnosed but hadn’t received antiparasitic treatment prior to this screening.

In strategy 2, six interventions were performed on site. Out of the 103 people who were screened in that strategy, it amounts to 17 people per intervention performed. 14 were diagnosed with *T. cruzi* infection (13.54% (IC95%: 10.10–28.24)). Nine of them were diagnosed for the first time. Three people received a full course treatment. Five people began periodic monitoring once they were diagnosed, and one of them had actually been prescribed a treatment before this screening but never started it.

No significant differences in the prevalence rate of *T. cruzi* infection diagnosis were found between the people screened in both strategies (Table 2).

**Table 2:** Comparison of prevalence rates of positive Chagas diagnosis among total screened participants by strategy

	**Total**		**Strategy 1**		**Strategy 2**				
	***N*** = 307		***N*** = 73		***N*** = 103		**RP (IC95%)**		***p***
	**% (IC95)**		**% (IC95)**		**% (IC95)**				
**Chagas prevalence rate**	**15.91**	(10.50—21.31)	**19.18**	(10.1- 28.24)	**13.59**	(6.96—20.23)	0.709	(0.359—1.398)	0.321
Adjusted Chagas prevalence rate *	15.91	(10.60—21.22)	**20.14**	(10.64—29.64)	**13.15**	(6.88—19.42)	0.653	(0.333—1.280)	0.215

*PR* Prevalence ratio. *IC95*%, Confidence interval of 95%. *P* *p*-value. ^a^Prevalence rate adjusted for age, sex, robust Poisson regression model

The socio-demographic characteristics of the people diagnosed with CD are shown in Table 3. Half of them were women, largely in the 50-and-over age group (46%) and residents of Barcelona (96%). The majority had stable employment (83%), had arrived in Spain five or more years before (84%), and had a Spanish national identity document (92%). All had an individual health card at the time of intervention. No significant differences were observed in any of the socio-demographic characteristics between the people diagnosed with CD in Strategy 1 and those diagnosed in Strategy 2.

**Table 3:** Characteristics of people diagnosed with Chagas disease

	**Total**	**Strategy 1**	**Strategy 2**	***p***
	***N*** = 28	***N*** = 14	***N*** = 14	
	***n*** (%)	***n*** (%)	***n*** (%)	
Socio-demographic characteristics				
**Gender**				1.00 a
Female	14 (50%)	7 (50%)	7 (50%)	
**Age**				
20–29	1 ( 4%)	0 ( 0%)	1 ( 7%)	0.71a
30–39	7 (25%)	4 (29%)	3 (21%)	
40–49	7 (25%)	4 (29%)	3 (21%)	
50 and over	13 (46%)	6 (43%)	7 (50%)	
**Country of origin**				
Bolivia	28 (100%)	14 (100%)	14 (100%)	
**Place of residence**				
Barcelona	25 (96%)	12 (100%)	13 (93%)	0.35
Other provinces	1 ( 4%)	0 ( 0%)	1 ( 7%)	
**Years from arrival in Europe**				
Less than 3 years	1 ( 4%)	0 ( 0%)	1 ( 9%)	0.15b
3 to 5 years	3 (12%)	3 (21%)	0 ( 0%)	
5 or more years	21 (84%)	11 (79%)	10 (91%)	
**Economic situation**				
Stable employment	20 (83%)	13 (93%)	7 (70%)	0.071b
Unstable employment	1 ( 4%)	1 ( 7%)	0 ( 0%)	
Unemployed	3 (13%)	0 ( 0%)	3 (30%)	
**Administrative situation**				
Spanish national identity document/foreigner identity number	22 (92%)	13 (93%)	9 (90%)	0.80b
Passport	2 ( 8%)	1 ( 7%)	1 (10%)	
Public healthcare system registration card*	27 (100%)	14 (100%)	13 (100%)	

^*^Dichotomous variable (yes/no). *P* *p*-value. a: contrast statistics calculated with Chi-squared test. b: contrast statistics calculated with Fisher’s exact test

### Results of infectious diseases screening at USIDVH

The second part of Fig. 1 describes the primary results of the general preventative screening.

In Strategy 1, all participants that chose to be screened for CD (73 total) took the infectious diseases screening simultaneously. Out of them, 51 people required and requested some type of medical attention for issues not related to CD. 39 people completed their vaccine schedule; 34 received a dose of the hepatitis B vaccine, 15 of the measles, mumps and rubella triple vaccine (MMR), three of the chickenpox vaccine, two of the tetanus-diphtheria vaccine, two of the hepatitis A vaccine, one of the SARS-Cov-2 vaccine, and one of the meningitis vaccine. 12 people were referred to other services (ten to their general practitioners, one to transcultural psychiatry, and one to endocrinology). 21 people (28.8%) in total were treated at USIDVH for parasite infection. The most frequent cases were of strongyloidiasis (8/73; 10.1%), giardiasis (4/73; 5.5%), eosinophilia (4/73; 5.5%), and syphilis (3/73; 4.1%).

In general, for Strategy 2, participants agreed to the general preventative screening at USIDVH if they tested positive for CD. In total, four underwent general screening, three completed their vaccine schedule (two hepatitis B and two MMR). Three required further treatment due to the results of the general screening; One was referred to their general practitioner and two were treated at USIDVH for infectious diseases (one strongyloidiasis and one chronic untreated Hepatitis C).

## Discussion

The results obtained highlight the strengths, advantages, and disadvantages of both strategies and emphasize the importance of addressing CD from a community perspective. Beecause the community health agents were Bolivians that had been trained on how to raise awareness and communicate about CD and spoke the participants’ languages and dialects, they were more likely to listen and receive information. This approach allowed the organized civil society to hold an active role identifying the main psycho-social triggers that should be addressed, as it encourages communities to participate in the design, development and set up of their own prevention and intervention strategies.

Besides, because the study took place within a consulate, where every employee was aware of the raising awareness campaign, we can assume that the location created a general trusting environment that eased the progress of the research perceived as safe and legitimate.

The study confirmed what prior evidence suggested: strategies designed for knowledge exchange and awareness raising alone have lower acceptance rates than strategies that also include an in-situ diagnostic test [15]. This is notably due to both logistical and psychosocial reasons. It is easier to have the blood test done directly on site the same day than to have to commute or drive to the health center some other day. Being screened on the spot also prevents overthinking about what the results might entail – a potentially positive diagnosis, the need to start a treatment, the awareness of being sick and the shame and stigma that go with it. However, it is important to highlight that the fact that some people agreed to be diagnosed at the health center demonstrates an improvement in those patients’ relationships with the healthcare system, most probably influenced by the trust in the community health agents and/or the members of the health team. That, as shown, also in turn facilitated the detection of other health conditions.

Strategy 2 has a strong geographical added value. Its participants mostly did not reside in the province of Barcelona. Compared to the “simple” sensitization strategies, that require less mobilization of healthcare personnel and resources (the intervention at the consulate depends solely on community health agents), *in-situ* screening strategies can have a greater geographic coverage because they enable to grant access to diagnosis to people living in further areas where healthcare is maybe less accessible. Said more simply, they help overcome geographic barriers to healthcare access.

The prevalence rates of CD observed in the screenings through both the IEC and in-situ outreach strategies were 19.18% (IC95%: 10.10–28.24) and 13.59% respectively. These rates are similar to those reported in previous studies: the Catalan Blood Bank found a *T. cruzi* seroprevalence infection rate of 10.2% in Bolivian donors [16]; in Spain a prevalence rate of 27.7% was found in the Bolivian population [20]; when at the European level the prevalence rate of CD was found to be 18.1% (IC95%: 13.9–22.7%) in residents of Bolivian origin [16].

An interesting take away from Strategy 1 is that screening people for CD appears as an opportunity to have them undergo an additional infectious disease screening. Offering a *T. cruzi* infection screening at the same time as screenings for other tropical diseases fits within the ethos of USIDVH and the logic of OSI that arise within the community [14]. Both strategies detected other healthcare needs—51 cases were treated for reasons unrelated to CD—among those who underwent the general preventative screening, mainly diagnosing infections and parasites (largely strongyloidiasis, 10.1% of cases). This is consistent with findings from other studies that highlight the most prevalent pathologies within the migrant population and specifically Bolivian population studies [18, 21]. This applies specifically to the case of strongyloidiasis. In a study by Salvador et al. [22] researchers found a prevalence rate between 5.5% and 26.8% in different cities, specifically 14% in Latin Americans. Another study carried out in Barcelona [22] found a prevalence rate of 16% in the Bolivian population; another in Alicante found a 12% prevalence rate in the Bolivian population [23]. Other cases (16.4%) from Strategy 1 needed medical attention from other services and were referred to general practitioners and, in two cases, psychiatry and endocrinology. Prior to the interventions, efforts have been made to open the dialogue among the different health system levels, and strengthen the process of patient referral and counter-referral. This partially explains the success of the patient care and must be a priority in future interventions.

In strategy 2, from those nine individuals who were referred to a healthcare setting after getting a diagnosis of CD in the in-situ test, only five attended the first appointment, preventing the appropriate access to healthcare and follow up. That, again, is intrinsically linked to the psycho-social barriers revolving around CD. Other studies have previously shown that repeated attempts to reschedule a second appointment can improve the attendance and follow-up of diagnosed patients [24].

It was also perceived that the many psycho-socio-cultural barriers, such as fear and stigma, that come with CD [3] also played a big role on whether or not people would agree to participate in the screening. For instance, it is no coincidence that in both strategies there was a higher prevalence of women, who make up the greater proportion of the Bolivian migrant population in recent decades [17]. This gender discrepancy in interest in participating in awareness and screening events related to CD has been highlighted on several occasions [12, 13, 19]. According to these previous studies, fear of death and knowledge of congenital CD transmission drives women to show more concern and interest in screening. They feel guilty, worried, and responsible for a potential transmission of the disease to their children [3]. From information exchanges with health professionals, it seems that men tend to prioritize work over healthcare. They feel vulnerable at the idea of being sick because of the social negative image and consequences it engenders [3]. The simple fact that all participants, thanks to their public healthcare registration card, could have had access to a diagnosis prior to this research further emphasizes the weight of the stigma and fear barriers around CD. In 2022, in Catalonia, among the total number of people of Bolivian origin registered in the Catalan Statistic Institute 59.6% were women and 41.4% men [24]. Strategy 2 reached more men than strategy, constituting an interesting take away for future studies.

These health community interventions at the primary health care level have been identified as key opportunities for systematic integration (OSI), during the recent pandemic [14]. OSI are strategies designed to address co-infection and co-morbidity between diseases have also presented favorable outcomes with other diseases and are increasingly implemented worldwide since 2007 [25–29]. Not only do they represent an economic gain, but they also enable to test concomitant infections specific to the group of study, which here was strongyloidiasis.

Finally, due to the nature of this study there are several limitations to the interpretation of the results. It is not possible to carry out an overall quantitative statistical comparison of the effectiveness and efficiency of each strategy; it is also not possible to accurately compare the prevalence rates obtained in each strategy. This is because: 1) there is no comparable denominator for both strategies, as the number of people invited to participate in each strategy is unknown—it is therefore impossible to know the initial acceptance rate of each individual intervention; 2) the sample size is very small; 3) a sizable quantity of socio-demographic information is missing from people who could not be contacted through phone interview.

## Conclusion

This research was made possible thanks to the interdisciplinary work between health, social and political institutions, namely the health facilities coordinators, community health agents, members of health professional teams specialized on community health, peer educators and the Bolivian authorities in Barcelona. That designed the framework fit to address the many psychological and socio-economic factors related to CD diagnosis.

This type of study should always take into account the local reality and needs, keeping in mind the barriers to an early diagnosis and a long-lasting follow up. While the in situ screening reached more people living outside of Barcelona, with a more vulnerable administrative situation, a screening in the health center may have allowed a more comprehensive preventive approach, care and a stronger cooperation with the different specialties of the health system. The geographical argument is an important take away for future research: if in situ screening is available, it should always be considered.

As the research progressed, the necessary resources—both human and logistics—to be more efficient became clear. The schedule for the interventions evolved according to the needs and findings of the health team but also to the possibilities of consulate authorities. The frequency of carrying out strategy 2 twice a month was based on the preliminary successful results. In the same vein, the idea to incorporate a systematic strongyloidiasis screening in strategy 2 came when it appeared that the infection rate was quite prevalent among the participants and that the tools to incorporate such OSI were available. Such screening has proven to be beneficial for the patient and as a consequence the USIDVH has implemented it systematically.

Both strategies confirmed that community action is a key pillar to apprehend the multidimensionality of CD and create a climate of trust with the targeted population. CHAs and peer educators have the ability to bridge the gap between the vulnerable population and the health professional team and hence extend access to healthcare. Being surrounded by peers (both people from the intervention team and the staff of the Bolivian consulate—including the consul himself) speaking the same languages with the same accent and wording fundamentally eased interactions between the parties. Intercultural communication has been determinant for education activities aiming at empowering communities and implicating people in the decisions regarding their healthcare.

In a nutshell, this opportunity for systematic integration in an official workplace of a foreign government fueled legitimacy in the eyes of the people that it targeted and made them more confident in the healthcare offered, contributing to improve early access to diagnosis, care and disease prevention in a non endemic area.

## Data Availability

The study involves participant data that contains potentially identifying and sensitive patient information, and public sharing may compromise participant privacy. Therefore, the Hospital Universitari Vall d’Hebron, Catalan Institute of Health has imposed restrictions on making the data publicly available. Data is available on request from Dr. Israel Molina, Director of the Programme on International Health of the Catalan Institute of Health – PROSICS (imolina@vhebron.net).
